# Gene expression profiling of connective tissue growth factor (CTGF) stimulated primary human tenon fibroblasts reveals an inflammatory and wound healing response in vitro

**Published:** 2011-01-08

**Authors:** Axel Seher, Joachim Nickel, Thomas D. Mueller, Susanne Kneitz, Susanne Gebhardt, Tobias Meyer ter Vehn, Guenther Schlunck, Walter Sebald

**Affiliations:** 1 Universitätsklinikum Würzburg, Abteilung für Molekulare Innere Medizin, Würzburg, Germany; 2 Institut für Tissue Engineering und Regenerative Medizin, Universitätsklinikum Würzburg, Würzburg, Germany; 3 Molekulare Pflanzenphysiologie und Biophysik, Julius von Sachs Institut, Universität Würzburg, Würzburg, Germany; 4 Microarray Core Unit, IZKF (Interdisziplinäres Zentrum für Klinische Forschung), Universität Würzburg, Würzburg, Germany; 5 Physiologische Chemie II, Biozentrum, Universität Würzburg, Würzburg, Germany; 6 Universitätsklinikum Würzburg, Augenklinik, Würzburg, Germany

## Abstract

**Purpose:**

The biologic relevance of human connective tissue growth factor (hCTGF) for primary human tenon fibroblasts (HTFs) was investigated by RNA expression profiling using affymetrix^TM^ oligonucleotide array technology to identify genes that are regulated by hCTGF.

**Methods:**

Recombinant hCTGF was expressed in HEK293T cells and purified by affinity and gel chromatography. Specificity and biologic activity of hCTGF was confirmed by biosensor interaction analysis and proliferation assays. For RNA expression profiling HTFs were stimulated with hCTGF for 48h and analyzed using affymetrix^TM^ oligonucleotide array technology. Results were validated by real time RT–PCR.

**Results:**

hCTGF induces various groups of genes responsible for a wound healing and inflammatory response in HTFs. A new subset of CTGF inducible inflammatory genes was discovered (e.g., chemokine [C-X-C motif] ligand 1 [*CXCL1*], chemokine [C-X-C motif] ligand 6 [*CXCL6*], interleukin 6 [*IL6*], and interleukin 8 [*IL8*]). We also identified genes that can transmit the known biologic functions initiated by CTGF such as proliferation and extracellular matrix remodelling. Of special interest is a group of genes, e.g., osteoglycin (*OGN*) and osteomodulin (*OMD*), which are known to play a key role in osteoblast biology.

**Conclusions:**

This study specifies the important role of hCTGF for primary tenon fibroblast function. The RNA expression profile yields new insights into the relevance of hCTGF in influencing biologic processes like wound healing, inflammation, proliferation, and extracellular matrix remodelling in vitro via transcriptional regulation of specific genes. The results suggest that CTGF potentially acts as a modulating factor in inflammatory and wound healing response in fibroblasts of the human eye.

## Introduction

The connective tissue growth factor (CTGF) belongs to the CCN (cyr61, ctgf, nov) protein family. The known six members of this family (CTGF, cysteine-rich angiogenic inducer 61 [Cyr61], nephroblastoma overexpressed gene [Nov], and WNT1 inducible signaling pathway protein 1-3 [WISP1–3]) share a high amino acid sequence homology and are composed of four domains (except WISP-2) [1,2], a insulin like growth factor-binding-domain, a von Willebrand factor type C domain, a thrombospondin1 domain, and a cystin knot motif. All motifs contribute to the broad range of different molecular and biologic functions by enabling interaction of CTGF with a large number of cytokines, cell surface receptors or proteoglycans. The contribution of CTGF to cell proliferation and extracellular matrix (ECM) remodelling [3,4], the molecular interaction with cytokines like bone morphogenetic proteins (BMPs) or transforming growth factor beta (TGF-β) [5] or the binding to proteoglycans like heparin [6] and different cell surface receptors like integrins [7-9] led to the notation of CTGF as a “multifunctional matrixcellular protein” in fibroblasts [10]. One of the most noted functions is its role as a transmitter in the activation of fibroblasts induced by TGF-β. In this context, CTGF is an immediate early gene and downstream mediator of TGF-β thereby playing a key role in biologic processes like wound healing or in pathological situations like fibrosis by promoting fibroblast proliferation, inducing ECM remodelling, and initiating myofibroblast differentiation [3,4,11].

The relevance of CTGF in the human eye has been studied, but only little is known about the effect of CTGF, in particular, which genes or biologic processes are affected by CTGF. But identification of CTGF on the protein level in different eye fluids, like aqueous humor [12] and tear fluid of healthy individuals [13], as well as the increase of CTGF levels in pathological situations correlates with its important role in proliferation and wounding also in the human eye. Conformingly, *CTGF* mRNA is expressed in corneal scar tissue and membranes in activated corneal fibroblasts, retrocorneal membranes, and subepithelial membranes, but not in control corneas [14]. A role of CTGF in the pathogenesis of conjunctival scarring in ocular cicatricial pemphigoid (OCP) also has been shown. Cultured fibroblasts isolated from the conjunctiva of patients with OCP showed elevated CTGF expression on mRNA and protein levels [15]. CTGF expression levels are also elevated in patient’s eyes having proliferative vitreoretinal diseases, such as proliferative diabetic retinopathy and proliferative vitreoretinopathy (PVR) [16]. The role of CTGF in scar formation after glaucoma filtering surgery (GFS) has been analyzed in a rabbit animal model. CTGF and TGF-beta were maximally expressed by day 5 after surgery and both proteins are present in the bleb tissues after GFS [17].

To identify genes which are regulated upon administration of CTGF, we analyzed the gene expression profile of control and hCTGF stimulated HTFs using affymetrix^TM^ oligonucleotide arrays. The results show the induction of a wound healing and inflammatory response in light of the upregulation of inflammatory cytokines, anti-apoptotic and proliferative genes, and components of the ECM.

## Methods

### Cell culture

Small tenon biopsy samples were obtained from standard intraocular surgery. The tenets of the Declaration of Helsinki were followed, and an institutional ethics committee approval had been granted. Primary human tenon fibroblasts (HTFs) were cultured as an expansion culture of the human tenon explants and were maintained in the logarithmic growth phase. For all experiments, only cells from passages 2 to 8 were used. Primary HTFs and the human cell line HEK293T were cultured in Dulbecco’s modified Eagle medium (Gibco Life Technologies, Karlsruhe, Germany) supplemented with 10% heat-inactivated fetal calf serum (Gibco Life Technologies), 100 U/ml penicillin, and 100 µg/ml streptomycin (Biochrom, Berlin, Germany).

### CTGF protein expression and purification

The cDNA encoding for full-length rhCTGF (aa 1–349; Swiss-Prot P29279) was cloned into the eukaryotic expression vector pCEP4 (Invitrogen, Darmstadt, Germany). HEK293T cells were transfected with the expression plasmid using lipofectamine^TM^ (Invitrogen) according to the protocol in the supplemental section. Stable clones were selected by adding hygromycin B (Invitrogen). For protein expression, selected clones were cultured in serum-free DMEM medium. Recombinant hCTGF protein was purified from the supernatant employing a three-step purification protocol. First, rhCTGF was isolated from the supernatant by heparin-sepharose chromatography using the specific affinity of the cystin-knot domain to heparin [6] subsequently followed by BMP-2 (bone morphogenetic protein 2) affinity [5,18] and S200 size exclusion chromatography. The purity of protein was analyzed by SDS–PAGE and western blot analysis.

### SDS–PAGE and western blot analysis

Protein samples were analyzed by SDS–PAGE using a 12% acrylamide gel and visualized by coomassie blue staining. For western blotting, proteins were separated by SDS–PAGE and subsequently transferred to nitrocellulose membrane (Whatman, Schleicher & Schuell, Dassel, Germany) using a semidry blotting chamber. The membrane was incubated in blocking buffer (5% BSA, 0.05% Tween-20 in 10 mM Tris, 100 mM NaCl, pH 7.4) for 1 h and then incubated with a polyclonal goat α-CTGF antibody (1:500 dilution in blocking buffer; Santa Cruz, Heidelberg, Germany) for 1 h. The membrane was washed (10 mM Tris, 100 mM NaCl and 0.05% Tween-20) three times and incubated with a secondary antibody (rabbit α-goat horseradish peroxidase conjugated; diluted 1:5000 in blocking buffer; Santa Cruz) for 1 h at room temperature. Detection was performed using enhanced chemiluminescence by the addition of ECL Plus reagents (Amersham-Pharmacia, Freiburg, Germany).

### Biosensor measurement

A BIACORE^®^2000 system (BIAcore, Freiburg, Germany) was used for all biosensor experiments. Biotinylated BMP-2 was immobilized to a streptavidin-coated biosensor CM5 chip at a density of about 500 resonance units (RU). BMP-2 used for these experiments was expressed and purified as described [19]. Sensorgrams of the CTGF-BMP-2 interaction were recorded at a flow-rate of 10 μl min^−1^ at 25 °C. The association phase was set to five and the dissociation phase to three min. After each cycle, the biosensor chip was regenerated with 4 M MgCl_2_. Binding affinities were calculated by fitting the kinetic data to a 1:1 Langmuir binding model using the BIAevaluation software 2.2.4. Binding constants were determined from two independent experiments using at least six different ligand concentrations. Standard deviations for the calculated KD-values were found to below 50%.

### Fibroblast proliferation assay

Cells were seeded at a density of 6,000 cells/cm^2^ using a 96 microwell plate and were allowed to adhere for 16 h. Cells were serum-starved for 24 h in DMEM (3% FCS) and subsequently stimulated for 48 h with different concentrations of rhCTGF (10–6,000 ng/ml). Cells growing in the presence of 10% FCS were used as a reference to show the maximal proliferation of HTFs under normal culture conditions. During the final 24 h 10 μl [^3^H]-thymidine (0.25 μCi; Amersham-Pharmacia) was added to each well. The cells were harvested on fiber mats (Scatron, Lierbyen, Norway) and thymidine incorporation was measured using a beta-radioactivity intelligent thin layer analyzer (RITA). All assays were performed in duplicates and repeated three times.

### Microarray hybridization

For RNA expression profiling, 1x10^5^ HTFs of three different donors were stimulated with 6 µg/ml rhCTGF for 48 h as described before. Total RNA was extracted from cells using the RNeasy Mini Kit according to the manufacturer's instructions (Qiagen, Hilden, Germany). RNA integrity and comparability was tested using a BioAnalyzer (Agilent, Santa Clara, CA). RNA integrity numbers (RIN) for the isolated RNAs ranged between 9.7 and 10, indicating excellent integrity. Reverse transcription, second-strand synthesis, clean-up of double-stranded cDNA and subsequent synthesis of biotin-labeled cRNA was performed according to Affymetrix protocols (One-Cycle cDNA synthesis Kit / IVT Labeling Kit; Affymetrix, Santa Clara, CA) starting from 2 μg of total RNA. From each sample 15 µg of adjusted cRNA were hybridized to a human Genome U133 Plus 2.0 GeneChip^®^ and visualized with a GeneChip Scanner 3000 (Affymetrix). The RNA expression profiles of the three different donors were analyzed separately.

### Data analysis

Data were analyzed using different R packages from the Bioconductor project. Signal intensities were normalized by variance stabilization [20]. The quality of all data sets was tested by density plot and RNA degradation plot. Statistical analysis for selection of differentially expressed genes was performed using the limma package (Linear Models for Microarray Analysis). The core algorithm of the limma package is an implementation of the empirical Bayes linear modeling approach of Smyth [21,22] that can be used for stable analysis even for smaller sample sizes.

### Real-Time reverse transcription polymerase chain reaction (RT–PCR)

For RT–PCR analysis HTFs were stimulated with 6 µg/ml rhCTGF for 48 h in a 24-well plate as described before. Total RNA was harvested using RNeasy spin columns (Qiagen) according to the manufacturer’s recommendations. First-strand cDNA was synthesized using M-MLV Reverse Transcriptase (Promega, Mannheim, Germany) at 42 °C for 1 h using 1 µg total RNA. RT–PCR was conducted in a 96-well microtiter plate using the iCycler instrument (Bio-Rad, Munich, Germany). A total reaction volume of 25 µl contained 50 ng of cDNA assuming 100% yield in the cDNA synthesis reaction), 0.4 µM of each primer, 240 µM dNTP mix (Invitrogen), 1× Taq polymerase reaction buffer (Promega), 2 Units Taq polymerase (Promega), FITC and SYBRgreen (Sigma-Aldrich, St. Louis, MO). Cycling conditions included an initial denaturation step at 95 °C for 3 min, followed by 40 cycles consisting of a 15 s denaturation interval and a one min interval for annealing and primer extension at 60 °C. The housekeeping gene hypoxanthine-guanine-phosphoribosyltransferase 1 (*HPRT1*) served as a normalization standard. Primer pairs used for RT-PCR are listed in Table 1.

**Table 1:** RT-PCR primer pairs.

**Name**	**Primer sequence**	**Melting temp (°C)**	**Product size (bp)**
*HPRT1* for	GACCAGTCAACAGGGGACAT	61.8	194
*HPRT1* rev	ACACTTCGTGGGGTCCTTTT	61.0	
*TNFα* for	CCCGAGTGACAAGCCTGTAG	62.0	151
*TNFα* rev	GAGGTACAGGCCCTCTGATG	62.0	
*IL1β* for	GGGCCTCAAGGAAAAGAATC	58.3	205
*IL1β* rev	TTCTGCTTGAGAGGTGCTGA	59.5	
*IL6* for	AGGAGACTTGCCTGGTGAAA	60.3	180
*IL6* rev	CAGGGGTGGTTATTGCATCT	58.6	
*IL8* for	AGGGTTGCCAGATGCAATAC	58.3	213
*IL8* rev	CCTTGGCCTCAATTTTGCTA	56.4	
*CXCL1* for	GAAAGCTTGCCTCAATCCTG	57.5	214
*CXCL1* rev	TCCTAAGCGATGCTCAAACA	56.7	
*CXCL6* for	GGGAAGCAAGTTTGTCTGGA	59.4	237
*CXCL6* rev	CTTTCCCCCACACTCTTCAA	59.8	

## Results

### hCTGF binds specifically to BMP-2

**Figure 1:**
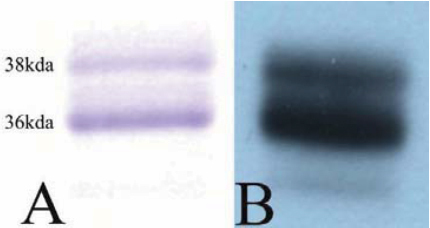
SDS–PAGE and western blot of rhCTGF. rhCTGF was expressed in HEK293T cells and purified by heparin and BMP-2 affinity chromatography. The purified protein (2 μg) was analyzed by (**A**) coomassie blue stained SDS–PAGE and (**B**) western blot using a α-CTGF antibody.

**Figure 2:**
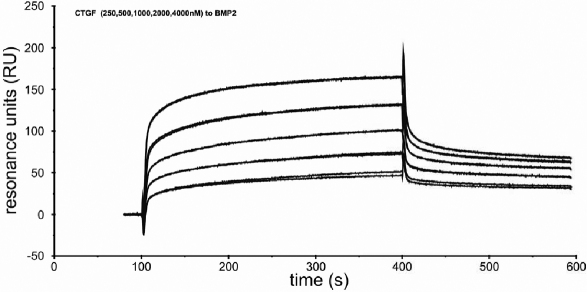
Sensorgrams of BIAcore measurements. Binding of CTGF at the indicated concentrations to immobilized BMP-2 was recorded at a flow-rate of 10 μl/min at 25 °C. The association phase was set to five and the dissociation phase to three min. Specific binding of hCTGF to immobilized BMP-2 reveals an apparent KD value of ~400 nM.

To study the biologic effects on human tenon fibroblasts (HTFs) recombinant hCTGF was prepared as described above. Purity and homogeneity of the protein was analyzed by SDS–PAGE and western blot (Figure 1A,B). Recombinant CTGF appears as two bands with an electrophoretic mobility corresponding to 36 kDa (unglycosylated) and 38 kDa (glycosylated isoform) in gel electrophoresis analysis as reported [4]. Biologic activity of recombinant hCTGF was tested by binding to immobilized BMP-2 using Biacore^TM^ technology. Specific binding of hCTGF to immobilized BMP-2 revealed an apparent *K*_D_ value of 400 nM consistent with previous results obtained for binding of CTGF [5] to the BMP-2 homolog BMP-4 (Figure 2).

### hCTGF induces proliferation in human primary tenon fibroblast

**Figure 3:**
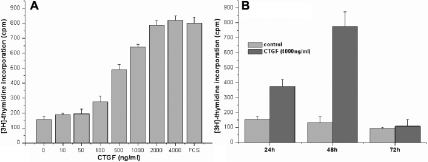
rhCTGF induced proliferation in primary tenon fibroblasts. HTFs were starved for 24 h and (**A**) subsequently stimulated for 48 h with increasing CTGF concentrations up to 4 μg/ml and 10% FCS as positive control and (**B**) for different time points between 24 and 72 h with a concentration of 4 μg/ml rhCTGF. In both experiments [3H]-thymidine DNA incorporation was measured after a 24 h incubation intervall by RITA.

In a second experiment the effect of hCTGF on cell proliferation was analyzed in a cell based assay measuring [3H]-thymidine incorporation into DNA. After 48 h hCTGF treated HTFs showed a fourfold increased DNA synthesis compared to negative control (Figure 3A). In all experiments, addition of hCTGF induced the same proliferation effect as observed for the FCS control. Additionally, a time series experiment was performed to show that the experimental setup chosen for gene expression analysis represents the optimal time point leading to a maximal proliferation rate. After 24 h HTFs exhibited slightly increased DNA synthesis (1.5 - twofold) with the peak levels after 48 h (3 - sixfold). In all experiments proliferation/DNA synthesis strongly decreased after 72 h with DNA synthesis in most cases being below the value for 24 h incubation (Figure 3B).

### Gene expression profiling of hCTGF stimulated human tenon fibroblasts

In the past ten years the relevance of CTGF for fibroblast biology with respect to proliferation, extracellular matrix production, wound healing, and diseases like fibrosis, has been shown [10,23,24]. Therefore we focused in our study on the relevance and influence of hCTGF on genes being differentially expressed in tenon fibroblasts of the human eye.

After CTGF stimulation for 48 h, 9.000 to 10.000 transcripts out of 18.400 analyzed transcripts were expressed. Of those a total of 110 transcripts are differentially expressed twofold or higher, for 21 of them the transcriptional difference exceeds more than threefold compared to the unstimulated control. Interestingly, the group of genes in fibroblasts showing upregulated expression upon hCTGF stimulation was significantly larger, with 92 transcripts compared to 18 genes being down-regulated. The strongest upregulation observed is 14.3 fold, the strongest down-regulation 3.1 fold compared to control cells. The ten most strongly up- and down-regulated genes are shown in Table 2 and Table 3.

**Table 2:** Top 10 upregulated genes in HTFs after hCTGF stimulation.

**Number**	**Gene title**	**Gene symbol**	**Ratio**	**p value**
1	chemokine (C-X-C motif) ligand 6	*CXCL6*	14.3	0.01
2	chemokine (C-X-C motif) ligand 1	*CXCL1*	9.4	0.15
3	interleukin 8	*IL8*	5.6	0.18
4	tumor necrosis factor, alpha-induced protein 6	*TNFAIP6*	5.0	0.18
5	interleukin 6 (interferon, beta 2)	*IL6*	4.9	0.18
6	serpin peptidase inhibitor, clade E	*SERPINE1*	4.7	0.05
7	chemokine (C-C motif) ligand 2	*CCL2*	4.3	0.05
8	kinesin family member 20A	*KIF20A*	4.1	0.19
9	interferon, alpha-inducible protein (clone IFI-6–16)	*G1P3*	3.9	0.18
10	ribonucleotide reductase M2 polypeptide	*RRM2*	3.3	0.37

**Table 3:** Top 10 down-regulated genes in HTFs after hCTGF stimulation.

**Number**	**Gene title**	**Gene symbol**	**Ratio**	**p value**
1	platelet derived growth factor D	*PDGFD*	−3.1	0.30
2	osteoglycin (osteoinductive factor, mimecan)	*OGN*	−3.0	0.08
3	osteomodulin	*OMD*	−3.0	0.23
4	chemokine (C-C motif) receptor-like 1	*CCRL1*	−2.7	0.24
5	SRY (sex determining region Y)-box 4	*SOX4*	−2.5	0.05
6	matrix Gla protein	*MGP*	−2.4	0.05
7	Ras association (RalGDS/AF-6) domain family 2	*RASSF2*	−2.2	0.23
8	selenoprotein P, plasma, 1	*SEPP1*	−2.2	0.18
9	alcohol dehydrogenase IB (class I), beta polypeptide	*ADH1B*	−2.2	0.41
10	v-maf musculoaponeurotic fibrosarcoma oncogene homolog B	*MAFB*	−2.2	0.15

To compose functional groups of genes allowing for a better insight into the biologic functions, the differentially expressed genes were analyzed and categorized with gene ontology (GO) [25]. The three main selection parameters of GO are molecular function, biologic process, and cellular component. The three groups can be subdivided into further functional subgroups, hierarchically classified and facilitate a better description of the biologic incidents. From the genes identified the 350 strongest up- and the 150 strongest down-regulated genes were selected and categorized via GO.

Mainly three categories are influenced: 1. Wound healing and inflammatory response; 2. Proliferation and anti-apoptosis; 3. Extracellular matrix remodelling. In the following we describe selected genes of each category or biologic process representative for each group.

### hCTGF initiate wound healing and inflammatory response in HTFs

**Figure 4:**
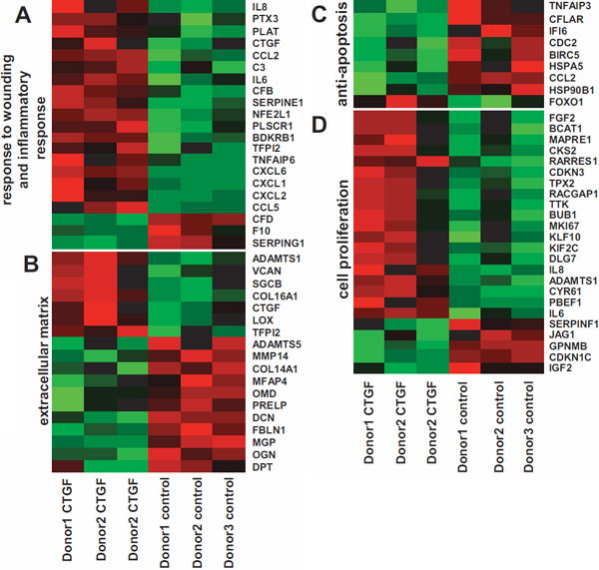
Heat map of differentially expressed genes in HTFs after CTGF stimulation. Differentially expressed genes were analyzed and categorized via “gene ontology.” The selected categories (**A**) “response to wounding and inflammatory response,” (**B**) “extracellular matrix,” (**C**) “anti-apoptosis,” and (**D**) “cell proliferation” are shown. Color represent measured signal intensity from lowest (green) to highest (red) value.

The GO analysis reveals mainly the initiation of a strong inflammatory and wounding response. Some of the strongest upregulated genes are members of the CC or CXC cytokine families, e.g., interleukin 6 (*IL-6*), interleukin 8 (*IL-8*), chemokine (*C-X-C motif*) ligand 6 (*CXCL6*), and chemokine (*C-C motif*) ligand 2 (*CCL2*; Figure 4A). The initial immune response is boosted by the elevated expression of complement cascade elements like complement factor B and complement component C3. Complement component C3 plays a key role in the activation of the complement system and is important for both, classical and alternative, complement activation pathways [26]. Also the elevated expression of the bradykinin receptor B1 indicates a wounding and inflammatory response. The receptor is usually de novo synthesized upon tissue injury and its activation is part of the inflammatory response [27].

**Figure 5:**
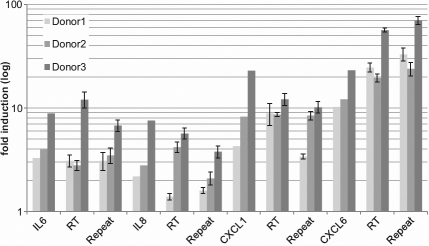
Expression level of inflammatory genes in human tenon fibroblasts after CTGF stimulation. HTFs were stimulated with 6 μg/ml CTGF for 48 h. Total RNA was isolated and gene expression was analyzed via affymetrix array. Selected genes out of the catagory “inflammation” were validated using real time RT–PCR. The figure shows the fold induction measured by affymetrix arrays compared to the results of real time RT–PCR (RT) for every single donor using the RNA used in array analysis. The results were additionally validated for every donor in an independent experiment (donors were stimulated again with CTGF). Isolated RNA was analyzed via RT–PCR (Repeat). RT–PCR was performed twice for every single experiment.

Array results were validated by analyzing six selected genes of the category “inflammatory and wounding response” (*TNF-alpha*, *IL-1*, *IL-6*, *IL-8*, *CXCL-1*, and *CXCL-6*). On the one hand we tested the RNA probes measured by affymetrix^TM^ oligonucleotide arrays and on the other hand we analyzed the RNA from one additional experiment in each case for all three donors. The array results could be confirmed for all six genes (Figure 5).

### CTGF induced genes mediate proliferation and anti-apoptosis in HTFs

Besides the wound healing and inflammatory response regulation and facilitation of cell proliferation was the strongest biologic activity found (Figure 4D). The proliferative effect of hCTGF in HTVs seems to be transmitted via two different ways. First, hCTGF induces a subset of genes directly responsible for proliferation and cell division by influencing cell cycle check points such as the two cyclins A2 und B1. Both promote cell proliferation [28,29] The ubiquitin conjugating enzyme E2C encodes a member of the E2 ubiquitin-conjugating enzyme family. It is also required for cell cycle progression [30,31].

Second, CTGF induces a survival program consisting of genes with anti-apoptotic properties (Figure 4C). As an example, Survivin is a member of the inhibitor of the apoptosis (IAP) gene family, which encodes negative regulatory proteins preventing apoptotic cell death [32]. The highest differentially expressed transcript out of this category is the interferon alpha-inducible protein 6 encoding the protein G1P3, which can restore the induction of apoptosis [33].

### Extracellular matrix is remodelled by hCTGF

The third strongly regulated group of genes addresses the ECM or genes that are involved in its modeling process (Figure 4B). Remarkably, a large number of these genes were down-regulated upon hCTGF stimulation. Some examples are decorin, dermatopontin, matrix Gla protein, osteomodulin and osteoglycin.

The lysyl oxidase belongs to the group of ECM genes being upregulated. It is an extracellular enzyme that initiates the cross-linking of collagens and elastin [34]. Another gene product, the tissue factor pathway inhibitor 2 (*TFPI-2*), is responsible for the suppression of matrix invasion activity [35] and for decreasing the amount of active matrix metalloproteinase 2 [36]. Interestingly, the CNN family members *Cyr61* and *CTGF* by itself seem also to be upregulated by an autocrine/paracrine positive feedback loop.

## Discussion

In the past decade different biologic functions were described for hCTGF. In fibroblasts TGF-β induced CTGF expression leads to cell proliferation, myofibroblast differentiation and extracellular matrix remodelling. These are some of the most prominent functions, implicating an important role of CTGF in biologic processes like wound healing or pathological situations like fibrosis [11]. Our results confirm the importance of CTGF also for primary HTFs in the biology of the human eye. The known CTGF-induced or -dependent processes are reflected by the genes identified in our affymetrix^TM^ oligonucleotide arrays. Mainly three biologic processes were shown to be influenced: 1. Wound healing and inflammation, 2. Cell proliferation, and 3. Extracellular matrix remodelling.

Besides the verification of established functions and the identification of new genes in these categories, mainly two aspects seem to be important. First, the expression of a subset of inflammatory genes is induced. Second, CTGF-induced genes playing normally a key role in bone formation also seem to be important.

In 2003 Wang et al. [37] described that administration of recombinant CTGF leads to an elevated DNA synthesis in primary porcine skin fibroblasts, but had no effect on inflammatory factors like IL-1, IL-6 or tumor necrosis factor alpha. In contrast, in our model system we could show that CTGF upregulates the transcription of various members of different cytokine families (e.g., *IL-6*, *IL-8*, *CXCL1*, *CXCL2*, or *CXL6*). This is of interest, because in diseases in which CTGF is involved, a strong inflammation reaction is often one factor that can promote a similar pathology like in fibrosis [38]. Another aspect is that the up-stream inducer of CTGF, TGF-β, is known to act anti-inflammatory under normal conditions. But under certain pathologic conditions, like cancer, this function is reversed and TGF-beta can enhance an inflammatory reaction of tissues or specific cell types [39]. In this Janus-like scenario CTGF might play an important role. Dependent on the context it promotes or inhibits the inflammatory response and is by this way a mediator of the TGF-β induced function and thereby a potential target for new therapy approaches. But why shows CTGF in human tenon fibroblasts an inflammatory potential?

One possibility might be due to the cell specificity, thus CTGF might exhibit different functions in fibroblasts of different origin. It has been shown that the cellular response to CTGF greatly depends on the cell type. In chondrocytes CTGF induces cell type specific genes like aggrecan and collagen X [40], whereas the gene collagen type I is not regulated. In vascular smooth muscle cells overexpression of CTGF reducedes proliferation rate and induces apoptosis [41], the contrary as observed in HTFs.

A second explanation for this phenomenon might be in the requirement of co-factors. It could be demonstrated for NRK cells that the induction of cellular responses by CTGF is co-factor dependent. CTGF alone did not show any effect in that cellular system, but in the presence of the epidermal growth factor (EGF), CTGF could promote cell proliferation. In combination with insulin like growth factor 2 (IGF-2), administration of CTGF leads to elevated collagen I synthesis and alpha smooth muscle actin expression [3].

We proofed this hypothesis for HTFs using Real Time RT–PCR by independent addition of both co-factors. We analyzed gene expression at different time points, but neither the addition of EGF nor IGF-2 had a significant effect on proliferation, collagen type I or alpha smooth muscle actin RNA expression (data not shown). However, it cannot be excluded that HTFs need a different specific factor to show a proliferative response.

On the other hand, an expression profile analysis with primary human skin fibroblast stimulated with the CTGF homolog Cyr61 is consistent with our gene expression results [42]. It could be demonstrated that also this angiogenic factor activates a genetic program for wound healing.Some of the genes induced by Cyr61 were also identified in our experiments (e.g., *PAI-1* or *uPA*). Interestingly, the expression of IL-1 beta was shown to be upregulated upon Cyr61 treatment. IL-1 beta can induce the expression of IL-6 and IL-8, thus also indicating an inflammatory potential of CTGF. Furthermore, similar to our results no regulation of alpha smooth muscle actin was observed after stimulation with Cyr61, although the importance of Cyr61 for alpha smooth muscle actin expression was shown in previous studies [43].

The discrepancies found for previous publications might be explained by the nature of CTGF as a “multifunctional matrixcellular protein.” The ability to interact with a broad range of different proteins enables CTGF to transmit opposite and cell-specific functions highly dependent on the context.

The second important observation is the regulation of genes in fibroblasts by CTGF, which are known to be involved in the process of bone formation. Osteoglycin is a small proteoglycan, which can interact and induce ectopic bone formation in concert with TGF-β [44]. Osteomodulin, also known as osteoadherin, is a small proteoglycan of the ECM and was shown to be induced by BMP-2 in the pre-myoblast cell line C2C12 [45]. The matrix Gla protein is as a powerful inhibitor of calcification of arteries and cartilage [46]. Two other important genes for bone formation also upregulated by CTGF are decorin and dermatopontin. Decorin synthesis is induced during conversion of myoblasts to osteoblasts in response to BMP-2 [47], while dermatopontin inhibits the BMP-2 induced expression of alkaline phosphatase (*ALP*) in C2C12 cells [48]. Furthermore dermatopontin is discussed to modify the binding properties of TGF-β to decorin [49].

Although the individual role of each of these genes in HRTs is yet unclear, their regulation by CTGF seems to be important under normal and pathological conditions. Using macroarray analysis Vittitow and Borras [50] investigated the consequences of high liquid pressure versus normal pressure treatment to cells to identify regulated genes of the trabecular meshwork. Interestingly, a group of genes being essential in cartilage and bone physiology was found to be regulated as well as the genes for matrix Gla protein and osteoglycin, which were also identified in our study as being regulated by CTGF. It is known that the application of pressure or mechanical forces influences cell morphology and leads to an upregulation of CTGF and subsequently to a change in the expression of responsive genes [51]. It is of great interest what roles these “bone specific genes” play in the context of fibroblast biology and pathology of the human eye.
